# Genetic variant located on chromosome 17p12 contributes to prostate cancer onset and biochemical recurrence

**DOI:** 10.1038/s41598-022-08472-x

**Published:** 2022-03-16

**Authors:** Anca Gabriela Pavel, Danae Stambouli, Ismail Gener, Adrian Preda, Gabriela Anton, Catalin Baston

**Affiliations:** 1 Molecular Genetics Department, Cytogenomic Medical Laboratory, Bucharest, Romania; 2 The Romania Academy, “Stefan S. Nicolau” Institute of Virology, Bucharest, Romania; 3 Department of Nephrology, Urology, Immunology and Immunology of Transplant, Dermatology, Allergology, Faculty of Medicine, “Carol Davila” University of Medicine and Pharmacy, Bucharest, Romania; 4 Department of Nephrology, Fundeni Clinical Institute, Bucharest, Romania; 5 Center of Urological Surgery, Dialysis and Renal Transplantation, Fundeni Clinical Institute, Bucharest, Romania

**Keywords:** Cancer genetics, Cancer prevention, Cancer screening, Urological cancer, Prostate cancer, Cancer genetics, Genetic markers, Genotype, Predictive markers, Prognostic markers, Cancer, Genetics, Biomarkers, Oncology, Risk factors, Urology, Prostate

## Abstract

The genetic contribution to prostate cancer (PC) onset and clinical heterogeneity has an important impact on the disease stratification accuracy. Despite the fact that radical prostatectomy (RP) is an effective treatment for localized PC, a considerable number of individuals develop biochemical recurrence (BCR) following surgery. In the present study, we decided to investigate the significance of genetic variability in a homogeneous group of Romanian men and to determine if genotyping could provide information regarding the possible implications of rs4054823 susceptibility loci in PC progression and outcome. A total of 78 samples from both PC and benign prostatic hyperplasia (BPH) patients were genotyped. The genotype frequencies were examined to see if there was a link between the 17p12 SNP and PC disease. When compared to the BPH group, the PC group had a significantly higher frequency of the T risk variant (*P* = 0.0056) and TT genotype (*P* = 0.0164). Subsequent analysis revealed that the TT genotype had a significantly higher frequency among younger PC patients based on their age at diagnosis and that it was related with a greater probability of BCR (*P* = 0.02). According to our findings, the TT genotype appears to be a risk factor for early-onset PC and a potential predictor for BCR after RP.

## Introduction

Prostate cancer (PC) (OMIM: 605095) is the second most common malignancy diagnosed among men, with a frequency of 29.3% and a mortality rate of 7.6% worldwide^1^. Moreover, the postoperative recurrence rate after radical prostatectomy (RP) remains approximately 20 to 40% within 10 years^2^. Currently, the prostate-specific antigen (PSA) biomarker is commonly used for the screening and detection of PC, but it is also an indicator for a number of benign (not cancerous) conditions, such as benign prostatic hyperplasia (BPH) (OMIM: 600082) and prostatitis (inflammation of the prostate)^3,4^. Post-treatment PSA serum levels are also measured for treatment failure identification.

In the case of RP, due to the complete removal of prostatic tissue, serum PSA levels are expected to be low or undetectable. Defining specific cutoff values to delineate PSA values after RP has been a subject of debate in a series of studies^5,6^. The European Association of Urology (EAU) and the American Urological Association (AUA) have recommended that the biochemical recurrence (BCR) be defined as serum PSA ≥ 0.2 ng/mL followed by a second confirmatory test with a PSA level greater than 0.2 ng/mL. However, Liu et al. revealed that because of a positive bias in PSA readings in a particular laboratory, patients were being incorrectly diagnosed with recurrent disease due to detectable PSA after prostatectomy when their PSA levels were actually undetectable^7^. To overcome the limitations of PSA testing, some studies suggest that genetic adjustment of PSA serum levels is needed^8^. Assessment of genetic risk factors may improve precision in diagnosis of PC. It is possible that in the future, particular genetic variants will be included in the screening protocols along with PSA blood tests and digital rectal examination, and they could be used as prediction factors for PC prognosis after RP.

The identification of patients who are at risk for BCR after surgical treatment plays an important role in selecting a personalized follow-up strategy to prevent clinical progression and cancer-related death^9^. Genome-wide association studies (GWASs) of PC patients and controls have led to the discovery of more than 100 single nucleotide polymorphisms (SNPs) that are associated with PC^10–17^. Furthermore, some of the genetic susceptibility loci have been shown to be correlated with disease aggressiveness^19,28–31,37^. By identifying patients with a high risk of more aggressive disease in the early stages, it possible to offer them an adjuvant therapy after RP that can improve the outcome by decreasing the risk for castration-resistant PC and cancer-specific death^18^.

This study focuses on a risk variant located on chromosome 17p12, rs4054823 SNP, which has been selected for analysis because it is the first reported SNP predisposing to aggressive PC^19^. The SNP rs4054823 resides in a region that is evolutionarily conserved but does not contain any known coding regions. The closest annotated gene is *HS3ST3A1*, a heparan sulfate biosynthetic enzyme that participates as a co-receptor in various growth factor families^20^. Alterations in heparan sulfate regulation have been shown to promote tumor growth and metastasis, which would correlate with a more aggressive cancer phenotype^21^. The aim of this study is to investigate the relation of 17p12 polymorphism with the onset and progression of PC in a clinical setting in a group of Romanian PC patients who underwent RP, as well as to determine whether this SNP can be considered as a predictive factor for postoperative BCR.

## Results

A total of 78 patients were included in this study. There were 50 (64.1%) patients with PC with a mean age 65.4 years, as well as 28 (35.9%) patients with BPH with a mean age 70.5 years. All the patients included in PC group underwent RP surgery. In all, 4 (8%) men had positive surgical margins (PSM). Of these, only two patients experienced BCR following local therapy. Table 1 shows the clinical and pathological data as the mean ± SD or number of subjects (percentage) for all patients included in the study.

**Table 1:** Analytical data of all participants in the study.

	PC, study group (*n* = 50)	BPH, control group (*n* = 28)	*P* value
*Age at diagnosis, years*			
Range	55–76	58–91	
Mean ± SD	65.4 ± 5.2	70.5 ± 7.2	0.011*
*PSA at diagnosis (ng/ml)*			
Range	4–73	6–16	
Mean ± SD	15.0 ± 5.0	11.0 ± 5.0	0.003*
*Clinical tumor stage (%)*		N/A	
T1	3 (6)		
T2	18 (36)		
T3	29 (58)		
*Lymph nodes (%)*		N/A	
N0	31 (62)		
N1	19 (38)		
*Gleason score (%)*		N/A	
6	1 (2)		
7 (3 + 4)	8 (16)		
7 (4 + 3)	15 (30)		
≥ 8	26 (52)		
*Biochemical recurrence (%)*		N/A	
No	22 (44)		
Yes	27 (54)		
Unknown	1 (2)		

*n*: number of subjects. SD: standard deviation. N0: there is no evidence of cancer in the regional lymph nodes. N1: cancer has spread to lymph nodes. N/A: not applicable. ^*^Significant difference at *P* < 0.05.

Table 2 shows the frequency of the rs4054823 SNP alleles and genotypes in patients with PC and BPH. Considering the CC genotype as the reference, the ORs of CT and TT genotypes for developing PC were statistically significant (*P* < 0.05). The frequency of the rs4054823 TT genotype in PC patients was significantly higher than in the BPH group (42% vs. 25%; *P* = 0.0164). In the PC group, there was a significant difference in frequency between the two alleles (C allele 36% and T allele 64%). Therefore, the TT genotype (T allele) could be considered a potential risk factor for PC.

**Table 2:** Genotype and allele frequency of rs4054823 in patients with PC and BPH.

	PC, %	BPH, %	*P* value	OR, *P*	95% CI
Genotypes			0.0164*		
CC	14	43		1	
CT	44	32		4.19, 0.0206	1.24–14.08
TT	42	25		5.14, 0.0112	1.45–18.22
Alleles			0.0056*		
C	36	33		1	
T	64	23		2.55, 0.0062	1.30–4.98

OR: odds ratio. CI: confidence interval. ^*^Significant difference at *P* < 0.05.

We evaluated the association between rs4054823 genotypes and clinical characteristics in the PC group. There was no significant association between the risk variant (TT genotype) and the aggressive disease (high PSA serum levels, high-grade score, advanced tumor stage, presence of lymph nodes). The results are illustrated in Table 3.

**Table 3:** Association between rs4054823 genotypes and clinicopathological characteristics. Significant difference at *P* < 0.05.

Genotypes	Tumor characteristics PSA serum levels (%)		*P* value
	Low	High	
CC	14.2	13.8	0.4706
CT	57.1	38.8	
TT	28.7	47.4	

Genotypes	Gleason grade (%)		*P* value
	Low-grade	High-grade	
CC	33.3	9.7	0.1736
CT	33.3	46.3	
TT	33.4	44.0	

Genotypes	Tumor stage (%)		*P* value
	Early	Advanced	
CC	23.8	6.8	0.2118
CT	42.8	44.8	
TT	33.4	48.4	

Genotypes	Lymph nodes (%)		*P* value
	Negative	Positive	
CC	19.3	5.2	0.3846
CT	38.7	52.6	
TT	42.0	42.2	

**Figure 1:**
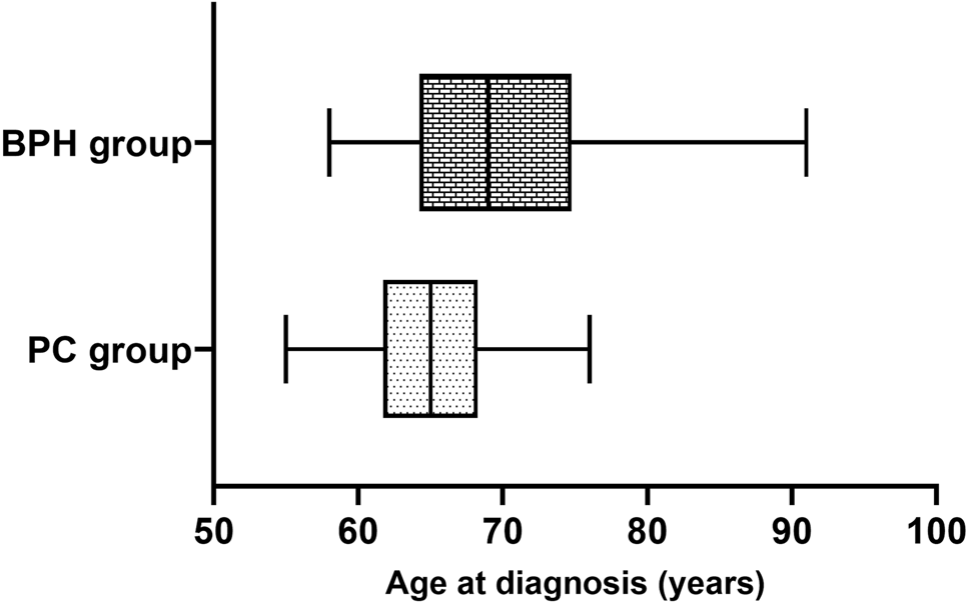
Differences between age at diagnosis in the PC and BPH groups.

The analysis revealed that PC patients were significantly younger at the time of diagnosis than individuals with BPH (*P* = 0.011), as shown in Fig. 1. According to age at diagnosis, we separated the cases into three different age groups: 50–59 years, 60–69 years, and ≥ 70 years. Table 4 shows the distribution of PSA serum levels according to age at diagnosis of PC. The highest PSA levels registered among all PC patients were found in the younger group (age 50–59 years) with a mean of 29.4 ng/ml, while the other two groups had 17.6 ng/ml and 18.7 ng/ml, respectively.

**Table 4:** PSA serum levels distribution according to age at diagnosis of PC.

Age	PSA ≥ 4 ng/ml	PSA, mean ± SD
50–59 years 60–69 years ≥ 70 years	7 (14%) 35 (70%) 8 (16%)	29.4 ± 22.4 17.6 ± 10.2 18.7 ± 12.4
Total	50	19.4 ± 13.0

SD: standard deviation.

**Figure 2:**
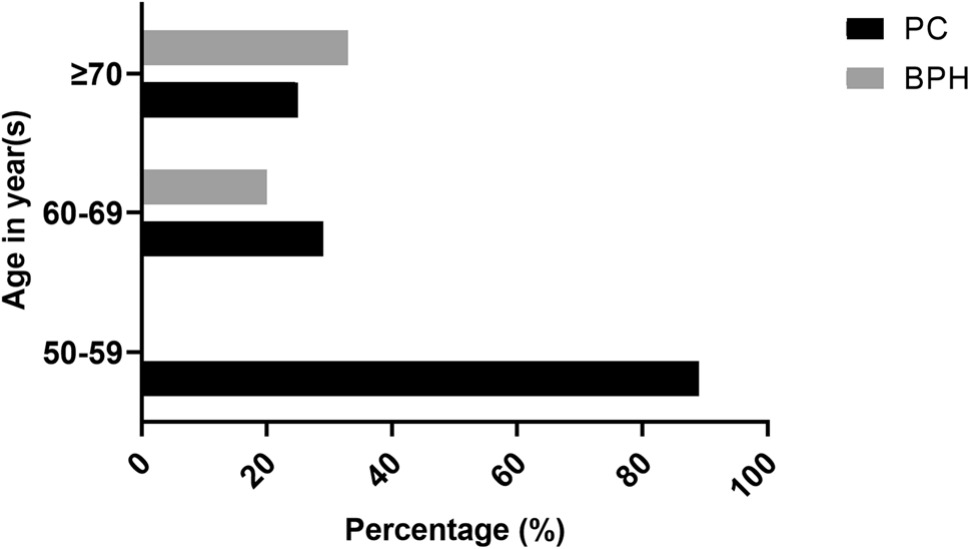
TT genotype distribution according to age at diagnosis within PC and BPH groups.

The mean age at diagnosis was compared for cases and controls. The onset age of PC patients with different genotypes varied significantly (*P* = 0.023). The mean age for patients carrying the TT genotype was significantly lower (62.9 ± 5.3 years) than in those with the CT and CC genotypes (66.2 ± 4.6 and 68.6 ± 4.9 years, respectively). No difference was observed among the three genotypes in the control group (*P* = 0.696). The age at diagnosis of PC and BPH was compared according to genotype, which showed a significantly higher frequency of the TT genotype in the younger PC group (*P* < 0.05). These results may suggest a possible contribution of the TT risk variant to the age at onset of PC (Fig. 2).

**Figure 3:**
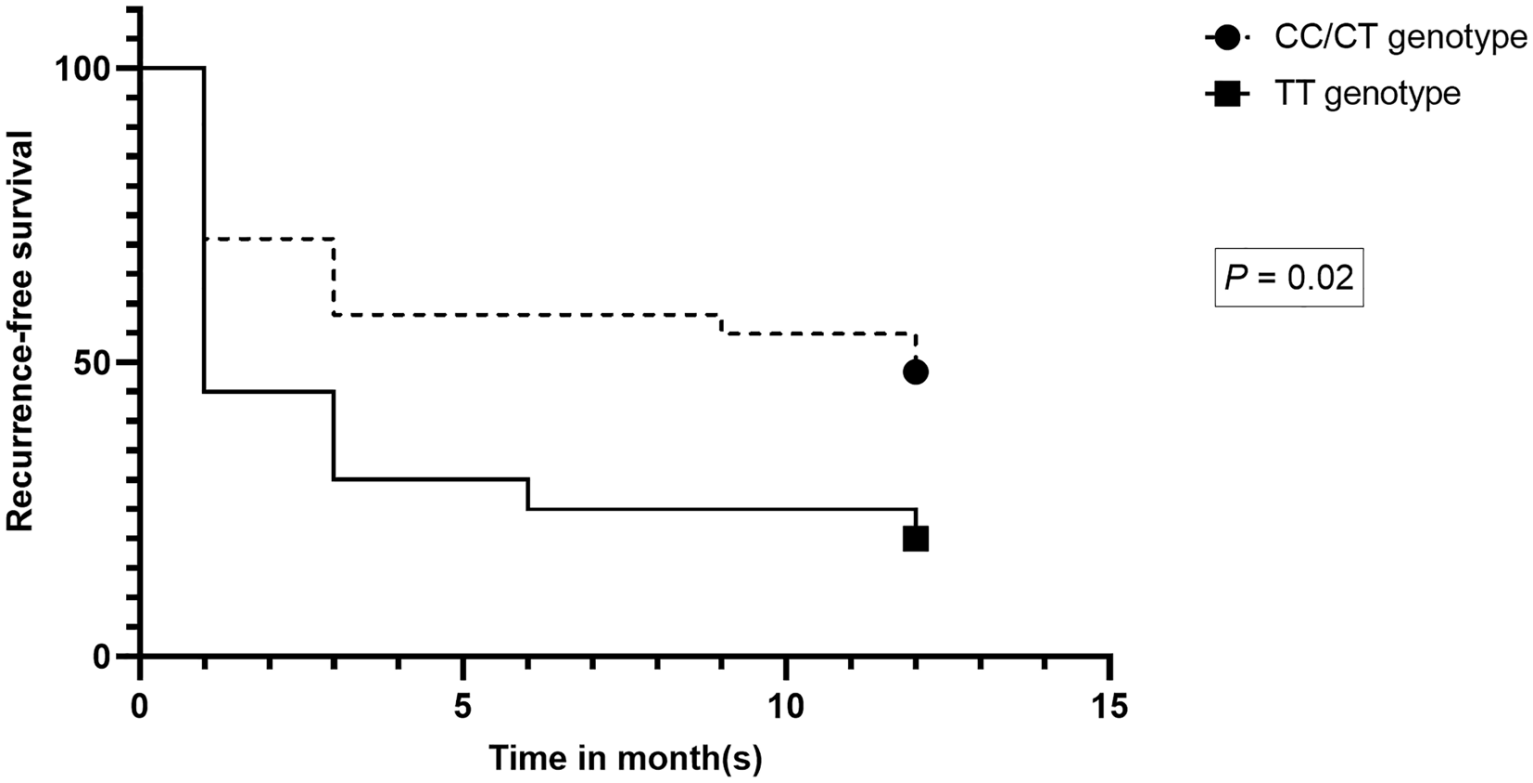
Kaplan–Meier survival curves of recurrence-free survival after RP according to 17p12 rs4054823 genotypes (TT vs CC/CT).

**Figure 4:**
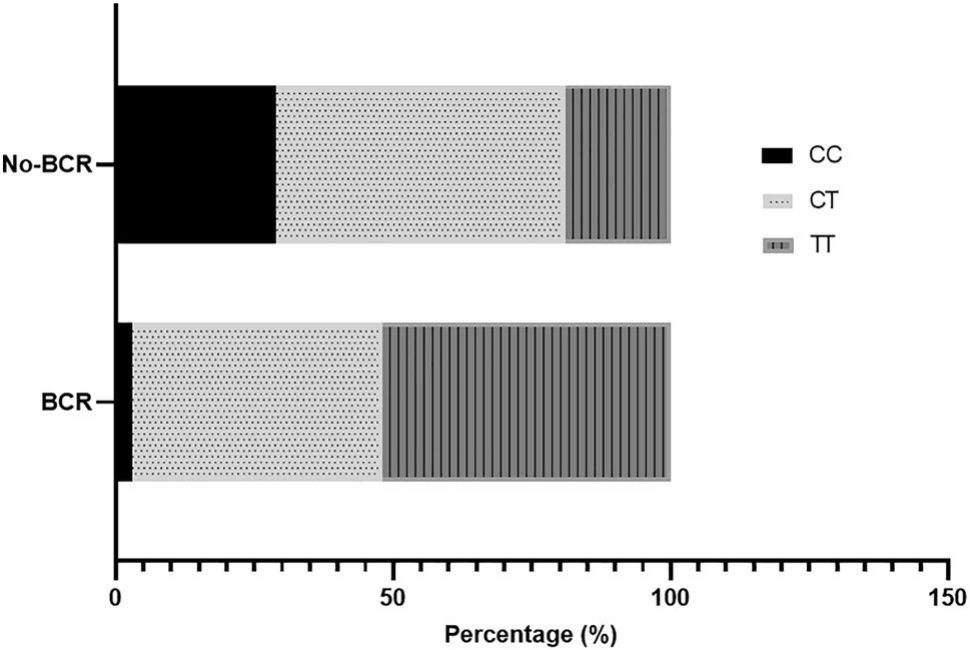
Distribution of rs4054823 SNP genotypes in cases of PC recurrence.

We investigated whether 17p12 polymorphism was associated with PC recurrence after RP, which showed a significant correlation of the TT genotype with an increased risk for BCR after RP in comparison to the CC and CT genotypes (Table 5 and Fig. 3). The distribution of rs4054823 genotypes among PC patients (Fig. 4) showed that the most frequent genotype found in the BCR group was the TT variant. In contrast, the group with no recurrent disease after RP had a lower frequency of TT genotype compared to the CC and CT genotypes, respectively.

**Table 5:** Association of 17p12 rs4054823 with BCR.

	No BCR, %	BCR, %	OR, *P*	95% CI
CC	18	10	1	
CT	65	42	1.16, 0.7321	0.48–2.76
TT	17	48	5.08, 0.0008*	1.96–13.14
CC versus CT/TT			1.96, 0.4098	0.39–9.79
CC/CT versus TT			4.45, 0.0230*	1.22-16.14

BCR: biochemical recurrence. OR: odds ratio. CI: confidence interval.

^*^Significant difference at *P* < 0.05.

## Discussion

Susceptibility to PC has a clear genetic component, as suggested by the approximately two-times higher risk among men with a family history of the disease^22^. Understanding the role of genetic alterations with respect to the occurrence and progression of PC to a lethal outcome in some patients has a decisive impact on the detection and prognosis of this type of cancer^23^. The recent discovery of various germline PC risk alleles potentially opens pathways for refining present predictive models. Although various GWASs have shown that some SNPs are associated with PC risk, it remains uncertain whether such genetic variants are also associated with the progression of the disease and whether these risk alleles can also predict the outcome of the PC treatment. Jeffers et al. highlighted that the discovery of possible predictors for PC recurrence after RP should facilitate decision strategies for post-RP PC disease management^24^.

There are several studies that have identified numerous genetic variants associated with PC risk and the aggressiveness of the disease^25–30^. In a review on the actual state of PC phenotype and GWASs, Pinto et al. showed that even though GWASs provide insight on new genetic information on the aggressive form of PC, there is still a need to implement approaches to confirm these findings in independent populations. They suggest that for better insight into the genetics of PC, it is important to use GWAS findings in clinical practice by introducing genetic testing for early detection of the disease. This would improve the stratification of individuals according to PC aggressiveness and the prediction of patient outcomes^31^.

The present study focused on an SNP located on chromosome 17p12 (rs4054823) that has been associated with PC progression and aggressiveness in previous studies^19,32^. However, these are retrospective studies examined patients selected from genetic analysis cohorts and frequently included individuals from different ethnic groups. In our study, we decided to explore the role of genetic variability in a homogeneous group of Romanian men to determine whether genotyping could provide information regarding the possible implications of rs4054823 susceptibility loci in the progression and outcome of PC in a clinical setting.

We reported the results of a case–control study that included the analysis of two groups from the viewpoint of a specific SNP, regardless of the effects of other genetic variations. The association between age as a risk factor and PC was investigated, and the results revealed that patients with PC were significantly younger than the BPH patients. It is known that the prostate gland becomes hyperproliferative in aging men and shows a tendency toward carcinoma. In this study, we found that aging is a risk factor for the abnormal growth of prostate tissue, which is mainly associated with benign tumors. This suggests that in this instance, the predisposition towards PC was also influenced by other factors, particularly genetic variability. The average age at diagnosis of our tested PC group was 65.5 years, which is in agreement with the average age for PC diagnosis in the US (currently 66 years)^33^.

According to the data distribution in these two groups, we examined the association between rs4054823 SNP and the overall PC risk. The results showed that patients with PC had a significantly higher frequency of the TT genotype and T allele of rs4054823 polymorphism compared to BPH patients. We also found that carriers of the homozygous TT genotype had more than five-times higher risk of PC. Similar results were found in a previous study conducted by Szulkin et al. on a large nationwide cohort of men treated for localized PC^32^. This study revealed that three of the 23 SNPs tested were associated with PC progression, including rs4054823 SNP on chromosome 17p12 (*P* = 0.008). In contrast, another study showed that the difference between TT risk genotype and the reference genotypes (CC and CT) of rs4054823 was not statistically significant (*P* = 0.617), and there was no association between PC and the risk variant of rs4054823^34^. Furthermore, a case–control study on a Romanian population conducted by Jinga et al. found no significant association between rs4054823 SNP and risk for PC (*P* = 0.226)^35^.

We also investigated whether the susceptibility loci on chromosome 17p12 also influenced clinicopathologic features of the disease. The results showed that the association of homozygous TT genotype and T allele with PSA serum levels, high-grade/stage tumor, and nodal invasion was not statistically significant (*P* > 0.05). Consequently, there was no significant association between the high-risk TT genotype and tumor aggressiveness. These results are in agreement with some of the earlier studies, which concluded there was no association between established PC risk variants and disease aggressiveness^28,32,36–38^.

The identification of a PSM after RP for PC is used as an indicator for surgical quality. The overall rates of PSM are between 6 and 22% in patients with localized PC treated with RP and vary according to disease characteristics, the length of follow-up data, and the surgical technique^39–41^. Retrospective studies have suggested that the presence of PSM is a risk factor for BCR^42,43^. Based on previous data, longer follow-up data will most likely show a higher BCR rate in patients with PSM^44^. Thus, PSMs have been associated with worse disease outcome in several studies, and most investigators consider this parameter as an independent predictor of BCR after RP^45^. Furthermore, previously reported data indicate that the impact of PSM on the risk of metastases or cancer-specific death might vary according to pathological features, such as the Gleason grade group, local stage, and the presence of seminal vesicle invasion or lymph node invasion^39,40,46–48^.

In the present study, the calculated PSM rate was 8% in patients with PC who underwent RP. The results are in line with previous studies^40^. Due to a small number of patients with PSM, of which only two experienced BCR, we could not correlate the presence of PSM with BCR in our group of patients. The influence of rs4054823 SNP genotypes on the age at diagnosis was examined, and the results revealed that the TT genotype had a higher frequency in the younger PC group (age 50–59 years) compared to the other two groups. These findings suggest that the T risk allele, particularly the TT genotype, can be associated with early onset of the disease and higher risk of PC.

The main challenge in PC care is improving the stratification of at-risk patients and the clinical outcomes. In this regard, we investigated whether this PC risk variant may influence the outcome of PC treatment, and we found that the TT genotype was significantly associated with the recurrence of PC in the case of RP. These results suggest a possible association between the TT genotype and biochemical recurrence of PC after RP, which may be of therapeutic significance.

According to GWASs, there are multiple novel loci for predisposition to PC. However, it is uncertain whether these SNPs are associated with the overall risk for PC or with recurrence of the disease after surgical treatment. Several studies tested whether particular SNPs selected from GWASs are associated with biochemical recurrence of PC and assessed the risk for metastatic disease. Gallagher et al. showed that rs61752561 in *KLK3* and rs2735839 in the *KLK2-KLK3* intergenic region were strongly associated with PC-specific survival and that rs10486567 in the *7JAZF1* gene was associated with BCR^49^. Ahn and colleagues studied the association between susceptibility loci and PC progression and recurrence, which revealed that SNPs in *MSMB* and 8q24 are associated with risk for metastatic PC, but they could not link any of this SNPs with the PC recurrence^50^.

Yajun et al. aimed to identify potential genes associated with PC recurrence following RP by using a comprehensive bioinformatic analysis of differentially expressed genes. The results of the analysis revealed that there are several crucial genes that may participate in PC recurrence^51^. Other studies regarding the association of genetic variants and the prostate-specific antigen recurrence disclosed that common SNPs that were corelated with tumor aggressiveness and disease progression can also be used to predict BCR in PC patients receiving RP^52–54^.

To our knowledge, the association of the rs4054823 genetic variant located on chromosome 17p12 and PC recurrence has not been evaluated previously. This study provides evidence that apart from the implication in the PC progression, the rs4054823 SNP can also be considered a potential risk factor for PC recurrence after RP. In the future, the genotyping of particular genetic variants might lead to a better stratification of PC patients, which could improve therapeutic strategies in patients with a high risk of recurrence.

There are several strengths to this study. The data used for the analysis were collected from a homogeneous group of Romanian patients in a clinical setting, and the results obtained were used to determine whether genotyping could provide information regarding the possible implications of rs4054823 susceptibility loci in the progression and outcome of PC. Nevertheless, the study had some limitations. The sample size was relatively small, and we had no information regarding family history or environmental factors that increase the risk for PC. The modest sample size, particularly when stratifying by age, limits our conclusions. However, our results could serve as a starting point for future investigations regarding the prevalence and clinical implications of germline variants for PC management.

Another limitation of our study could be related to the characteristics of the control group. Patients included in the control group were diagnosed and treated for BPH according to current urological guidelines (EAU and AUA). The clinical examination of the prostate based on DRE and serum PSA levels at the time of diagnosis did not indicate the presence of PC. Moreover, the postoperative histopathological result did not show any elements of malignancy or relapse of the disease during the periodic follow-up. Nevertheless, the risk for the presence of PC in the unsampled prostate of BPH patients cannot be excluded.

In summary, we have provided further evidence that the rs4054823 risk polymorphism located on chromosome 17p12 may be linked to PC. Our findings suggest that the TT genotype can be considered a potential risk factor for the onset of PC at a younger age. Moreover, our results suggest that the TT genotype is a potential risk factor for the biochemical recurrence of PC after RP. However, due to the limitations of our study, the results need to be confirmed by further molecular studies on larger cohorts. Finding the optimal treatment plan for PC patients, especially those with recurrent disease, represents a clinical challenge. Therefore, in addition to potential clinical benefits, identification of inherited genetic variants associated with PC progression and outcome may extend our knowledge regarding the underlying cause PC, providing insight into possible preventive and therapeutic targets.

## Materials and methods

### Study subjects

Peripheral blood samples were collected from 78 patients in tubes containing ethylene diamine tetra acetic acid (EDTA). Of the 78 patients, 50 had been diagnosed with PC, and 28 patients had been diagnosed with BPH. The patients were selected between 2014 and 2016 from the Fundeni Clinical Institute, Bucharest, ROU. The patients were divided into two groups to determine whether there is an association between 17p12 SNP rs4054823 and prostate carcinoma. The PC patients were the study group, and patients diagnosed with BPH were considered as the control group.

The diagnosis of prostate carcinoma was confirmed by clinical and laboratory examination. To determine whether the rs4054823 risk genotype can influence the age of PC onset, we divided the patients into three different age groups: 50–59 years, 60–69 years, and ≥ 70 years. To assess disease progression after RP, PSA was determined for all PC patients who underwent surgery. The measurements were performed at 1, 3, 6, 9, and 12 months after surgery. All cases that had a rising serum PSA level greater than 0.2 ng/mL followed by confirmatory PSA were considered for recurrent disease.

The study has been approved by the Ethics Committee of the Fundeni Clinical Institute (Bucharest, Romania) and was performed in accordance with the Declaration of Helsinki. Written informed consent was obtained from all participants before sample collection. All methods were performed in accordance with relevant guidelines and regulations. Standard protocols were followed to ensure the confidentiality of personal data.

### Genotyping

Germline DNA from the PC patients and the control group was isolated from whole blood samples using the Wizard Genomic DNA Purification Kit (Promega, Madison, WI). The DNA quantity was determined using a Qubit 3.0 Fluorometer (Thermo Fisher Scientific, Carlsbad, CA). Post-extraction quality control was performed using spectrophotometry and 1% agarose gel.

Genotyping of the rs4054823 C/T polymorphism located on chromosome 17p12 was performed by allelic discrimination with Taqman 5’-nuclease assays (Thermo Fisher Scientific, Carlsbad, CA) according to the manufacturer’s recommended protocols. The reaction was carried out in a 10-µl final volume containing 1–5 ng of each purified gDNA sample on a StepOne Real-Time PCR system (Thermo Fisher Scientific, Carlsbad, CA). The following thermal cycling conditions were used: an initial step at 95 °C for 10 min, followed by 40 cycles of 15 s at 95 °C and 1 min at 60 °C. The obtained data were analyzed using the automated SNP genotype calling available with the StepOne system software.

### Statistical analysis

Clinical data are presented as the mean ± standard deviation (SD) for continuous variables and as numbers and percentages for categorical variables. The Mann–Whitney U test was used to compare differences between the two groups (PC and BPH) according to age at diagnosis. Allelic and genotype frequencies in the cases and controls were calculated and tested through Fisher’s exact test. Correspondingly, other analysis was conducted by comparing age at diagnosis for the TT risk-genotype.

The mean age at diagnosis was compared for both PC and BPH groups according to genotypes using an ANOVA test. Fisher’s exact test was used to evaluate the frequency distribution of risk alleles and genotypes in relation to clinico-pathological features (nodal invasion, tumor stage, PSA serum levels, and Gleason score). Logistic regression was used to evaluate the diagnostic and prognostic value of the TT genotype in relation to clinic-pathological parameters. The significance of the rs4054823 SNP genotype as a predictor for BCR was determined using Kaplan–Meier analysis. Odds ratios (ORs) were estimated as a measure of relative risk of PC with their 95% confidence intervals (CIs).

In all analyses, a two-tailed *P* values of less than 0.05 were considered as statistically significant. GraphPad Prism 9 software was used for all statistical analyses of data. Furthermore, power analysis was performed with G*Power 3.1 software. A post-hoc method was used. Given a significance level of 0.05 and sample sizes of 50 PC patients and 28 BPH patients, the calculated effect size was 0.86, and the power of the study was found to be more than 80%.

### Ethics approval and consent to participate

The present study was approved by the Ethics Committee of the Fundeni Clinical Institute (Bucharest, Romania). All participants provided written informed consent.

## Data Availability

All data generated or analyzed during this study are available from the corresponding author on reasonable request.
